# A comparison of computational methods for detecting bursts in neuronal spike trains and their application to human stem cell-derived neuronal networks

**DOI:** 10.1152/jn.00093.2016

**Published:** 2016-04-20

**Authors:** Ellese Cotterill, Paul Charlesworth, Christopher W. Thomas, Ole Paulsen, Stephen J. Eglen

**Affiliations:** ^1^Cambridge Computational Biology Institute, University of Cambridge, Cambridge, United Kingdom; and; ^2^Department of Physiology, Development and Neuroscience, Physiological Laboratory, University of Cambridge, Cambridge, United Kingdom

**Keywords:** bursts, spike trains, computational methods, stem cells, development

## Abstract

Accurate identification of bursting activity is an essential element in the characterization of neuronal network activity. Despite this, no one technique for identifying bursts in spike trains has been widely adopted. Instead, many methods have been developed for the analysis of bursting activity, often on an ad hoc basis. Here we provide an unbiased assessment of the effectiveness of eight of these methods at detecting bursts in a range of spike trains. We suggest a list of features that an ideal burst detection technique should possess and use synthetic data to assess each method in regard to these properties. We further employ each of the methods to reanalyze microelectrode array (MEA) recordings from mouse retinal ganglion cells and examine their coherence with bursts detected by a human observer. We show that several common burst detection techniques perform poorly at analyzing spike trains with a variety of properties. We identify four promising burst detection techniques, which are then applied to MEA recordings of networks of human induced pluripotent stem cell-derived neurons and used to describe the ontogeny of bursting activity in these networks over several months of development. We conclude that no current method can provide “perfect” burst detection results across a range of spike trains; however, two burst detection techniques, the MaxInterval and logISI methods, outperform compared with others. We provide recommendations for the robust analysis of bursting activity in experimental recordings using current techniques.

## Abstract (precis)

*We provide an unbiased quantitative assessment of eight existing methods for identifying bursts in neuronal spike trains. We reveal limitations in a number of commonly used burst detection techniques and provide recommendations for the best practice for accurate identification of bursts using existing techniques. An analysis of the ontogeny of bursting activity in a novel data set of recordings from human induced pluripotent stem cell-derived neuronal networks, using the highest-performing burst detectors from our study, is also presented*.

## NEW & NOTEWORTHY

*We provide an unbiased quantitative assessment of eight existing methods for identifying bursts in neuronal spike trains. We reveal limitations in a number of commonly used burst detection techniques and provide recommendations for the best practice for accurate identification of bursts using existing techniques. An analysis of the ontogeny of bursting activity in a novel data set of recordings from human induced pluripotent stem cell-derived neuronal networks, using the highest-performing burst detectors from our study, is also presented*.

the tendency of neurons to fire brief periods of spikes in quick succession, or bursts, has been observed extensively both in vitro and in vivo (Pasquale et al. 2010; Weyand et al. 2001). Bursting is believed to be associated with a variety of physiological processes, such as synapse formation (Maeda et al. 1995) and long-term potentiation (Lisman 1997). With the use of recordings of the electrical activity of neurons cultured on microelectrode arrays (MEAs), various aspects of in vitro network activity, including bursting, can be readily examined. MEAs have thus been used to study changes in the spontaneous activity patterns exhibited by neuronal networks over development (Charlesworth et al. 2015; Wagenaar et al. 2006). Analysis of bursting activity has also been used as an important tool in applications such as studying the impact of genetic or chemical manipulations on network activity (Charlesworth et al. 2016; Eisenman et al. 2015).

Despite the prevalence of bursting as a feature used to study neuronal network activity, the concept of a burst still lacks a definitive formal definition (Cocatre-Zilgien and Delcomyn 1992; Gourévitch and Eggermont 2007) or a single widespread technique used for detecting bursts. Instead, a variety of burst detectors exist, many of which have been developed and verified by researchers on an ad hoc basis using specific data sets and singular experimental conditions.

One common approach to burst detection is identification of periods of bursting using simple thresholds, which impose limits on values such as the minimum firing rate or maximum allowed interspike interval (ISI) in a burst. These thresholds can either be fixed values (Chiappalone et al. 2005; Mukai et al. 2003) or derived from properties of the spike trains, such as the mean ISI (Chen et al. 2009), total spiking rate (Pimashkin et al. 2011), or some form of the distribution of ISIs or discharge density (Bakkum et al. 2013; Cocatre-Zilgien and Delcomyn 1992; Kaneoke and Vitek 1996; Kapucu et al. 2012; Pasquale et al. 2010; Selinger et al. 2007). Another type of burst detection techniques is the “surprise-based” methods, which detect bursts as deviations from an assumed underlying firing rate distribution (Gourévitch and Eggermont 2007; Ko et al. 2012; Legéndy and Salcman 1985). There are also a variety of methods based on some variation of these ideas, or which take other approaches entirely (Hennig et al. 2011; Tam 2002; Tokdar et al. 2010; Turnbull et al. 2005; Wagenaar et al. 2005; Weihberger et al. 2013; Xia et al. 2003).

Most existing studies involving analysis of bursting activity in MEA recordings have been performed on experimental data from rodents (Charlesworth et al. 2015; Mazzoni et al. 2007). In recent years, it has been demonstrated that networks of neurons derived from human stem cells can be grown successfully on MEAs and exhibit spontaneous electrical activity, including bursting behavior (Heikkilä et al. 2009; Odawara et al. 2016). These networks often exhibit far more variable bursting activity than more commonly studied rodent neuronal networks (Kapucu et al. 2012). There is thus a demand for the development of robust standardized analysis methods for identifying bursts in such networks.

Here we have reviewed eight existing burst detectors, selected to encompass a range of contemporary burst analysis methods, and evaluated their effectiveness at detecting bursts, particularly in spike trains with properties resembling those of human stem cell-derived neuronal networks. Each burst detector was used to analyze bursts in synthetic spike trains and in vitro MEA recordings from mouse retinal ganglion cells (RGCs). This allowed for a quantitative assessment of the performance of each method in a variety of contexts. On the basis of these results, we offer suggestions to researchers regarding the best approaches for comprehensive burst analysis. The highest-performing methods in our study were also used to describe the ontogeny of bursting activity in networks of human induced pluripotent stem cell (hiPSC)-derived neurons over several months of development.

## MATERIALS AND METHODS

### Burst Analysis Methods

Eight burst detectors that we believed to be representative of the major approaches to burst detection and that have sufficient general applicability to allow for their use on a variety of spike trains were chosen for analysis. Other methods were excluded from the analysis for a variety of reasons, including that they do not explicitly identify the location of bursts in a spike train (van Elburg and van Ooyen 2004) or we believe that they have been superseded by more refined methods (Chiappalone et al. 2005; Selinger et al. 2007).

A brief description of each of the eight burst detectors applied to a single spike train is given below, and we refer the reader to the original sources for detailed descriptions. Where possible, we reused existing code from the original authors to implement each method. All analyses presented here were performed with R statistical software (R Core Team 2015), and the code used to implement each burst detector is publicly available at https://github.com/ellesec/burstanalysis.

In the implementation of each method, the minimum number of spikes in a burst was set to three and other parameters were left set to the standard parameters suggested by the authors (Table 1). The exception to this was the three surprise-based burst detectors, for which the minimum surprise value was set to −log(0.01) for all three methods for consistency.

**Table 1.** The eight burst detectors and the parameter values used for the implementation of each method on synthetic spike trains

	Parameter	Value
Poisson surprise (Legéndy and Salcman 1985)	Minimum surprise value	−log(0.01) ≈ 4.6
MaxInterval (Nex Technologies 2014)	Maximum beginning ISI	0.17 s
	Maximum end ISI	0.3 s
	Minimum interburst interval	0.2 s
	Minimum burst duration	0.01 s
	Minimum spikes in a burst	3
Cumulative moving average (Kapucu et al. 2012)	α_1_ (α_2_)	1.0 (0.5) if skew < 1
		0.7 (0.5) if 1 ≤ skew < 4
		0.5 (0.9) if 4 ≤ skew < 9
		0.3 (0.1) if 9 ≤ skew
Rank surprise (Gourévitch and Eggermont 2007)	Largest allowed ISI in burst	75th percentile of ISIs
	Minimum surprise value	−log(0.01) ≈ 4.6
ISI rank threshold (Hennig et al. 2011)	Rank threshold, θ_*R*_	0.5
	Spike count cutoff, θ_*C*_	*C* such that *P*(*C*) = 0.05
Robust Gaussian surprise (Ko et al., 2012)	Minimum burst surprise	−log(0.01) ≈ 4.6
LogISI (Pasquale et al. 2010)	Maximum cutoff value	100 ms
Hidden semi-Markov model (Tokdar et al. 2010)	Probabilistic cutoff	0.5
	Other parameters (*n* = 23)	As per paper*

* These parameters were left set to the default values provided in the “burstHSMM” R package.

#### LogISI method (Pasquale et al. 2010).

Bursts are detected using the histogram of the log-adjusted ISIs on a spike train. The peaks of this histogram are found using a tailor-made peak finding algorithm outlined in Pasquale et al. (2010), and the largest peak corresponding to an ISI of ≤100 ms is set as the intraburst peak. In the absence of such a peak, no bursts are found. The minimum values between the intraburst peak and all subsequent peaks are found, and a void parameter, which represents how well the peaks are separated, is calculated for each minimum. The ISI value corresponding to the first minimum at which the void parameter exceeds a threshold value of 0.7 is set as the cutoff value for burst detection, maxISI. Bursts are then detected as any series of three or more spikes separated by ISIs smaller than maxISI. If no cutoff is found, or if maxISI > 100 ms, bursts are found with a 100-ms cutoff, and then extended to include any spikes within maxISI of the edges of each burst.

#### Cumulative moving average method (Kapucu et al. 2012).

The cumulative moving average (CMA) of the histogram of ISIs is calculated. The skewness of this CMA distribution is used to determine the values of two parameters, α_1_ and α_2_, according to the scale given in Kapucu et al. (2012) and shown in Table 1. The ISI value of the histogram bin at which the CMA is closest in value to α_1_·CMA_MAX_ is set as maxISI, where CMA_MAX_ is the peak of the CMA distribution. Again, bursts are defined as sequences of more than two spikes separated by ISIs less than maxISI.

Kapucu et al. (2012) also suggest expanding these bursts to include burst-related spikes, which are found with a cutoff set at the histogram bin at which the CMA is closest to α_2_·CMA_MAX_. Any spikes within this cutoff distance from the beginning or end of the original bursts are classified as burst-related spikes. However, for our purposes, we only examined the original burst cores detected from this method and omitted any burst-related spikes.

#### ISI rank threshold method (Hennig et al. 2011).

The rank, *R*(*t*), of each ISI relative to the largest ISI on the spike train is calculated. A rank cutoff, θ_*R*_, is chosen, and a spike count cutoff, *θ*_*C*_, is calculated from the distribution of spike counts over 1-s intervals on the spike train. A burst begins at a time *t* if the spike count over the following second exceeds θ_*C*_, and its subsequent ISI satisfies *R*(*t*) < θ_*R*_. The burst continues until the spike count over the following 1-s interval falls below θ_*C*_/2.

#### Poisson surprise method (Legéndy and Salcman 1985).

The baseline firing rate on a spike train is assumed to follow a Poisson process with rate λ equal to the mean firing rate over the entire train. The Poisson surprise (PS) statistic for an interval of length *T* containing *N* spikes is defined as

S=−log(p)

where *p* is the probability of *N* or more spikes randomly occurring in an interval of length *T* in the underlying Poisson process. Bursts are chosen so as to maximize the PS statistic over the entire spike train with a surprise maximization algorithm outlined in Legéndy and Salcman (1985), and any bursts with a PS value below a threshold significance level are discarded.

#### Rank surprise method (Gourévitch and Eggermont 2007).

The ISIs on the spike train are ranked, with the smallest ISI given a rank of 1. For each possible bursting interval, the rank of all ISIs on the interval are summed, and the probability, *p*, of a value of equal or lesser value being drawn randomly from a discrete uniform sum distribution is calculated. Bursts are chosen so as to maximize the rank surprise (RS) statistic, defined as RS = −log(*p*), across the entire spike train, and any bursts with a RS statistic below a predefined significance threshold are discarded.

#### Robust Gaussian surprise method (Ko et al. 2012).

Bursts are regarded as outliers from a “central distribution” of ISIs, which is estimated from the distribution of normalized log(ISIs) on a spike train. ISIs are considered to be potentially within bursts if they lie below −2.58 times the median absolute deviation of this distribution. For each potential burst, the robust Gaussian burst surprise value, GS_B_ = −log(*p*), is calculated, where *p* is the probability that the sum of the normalized log(ISIs) in the interval is less than or equal to the sum of an equivalent number of i.i.d. Gaussian random variables with mean and standard deviation equal to those of the central distribution. These initial bursts are then extended to include surrounding spikes until the maximal surprise value is found. Finally, any bursts with a surprise value below a predefined significance threshold are discarded.

#### Hidden semi-Markov model method (Tokdar et al. 2010).

Neurons are assumed to stochastically alternate between a “nonbursting” and a “bursting” state, labeled *states 0* and *1*, respectively. Spiking activity is modeled with a hidden semi-Markov model (HSMM), with transition times between the two states modeled as two gamma distributions, *f*_0_^ITI^ and *f*_1_^ITI^. Within each of the two states, the distribution of ISIs are modeled using two additional gamma distributions, *f*_0_^ISI^ and *f*_1_^ISI^. The parameters of these four distributions are learned from the data. Under these assumptions, the posterior probability that the neuron is in a bursting state at any given time is calculated with a Markov chain Monte Carlo (MCMC) method, and any periods in which this probability exceeds a threshold value are classified as bursts. An R package to implement this method is available at https://stat.duke.edu/~st118/Software/.

#### MaxInterval method (Nex Technologies 2014).

Any series of consecutive spikes fulfilling five threshold parameters, the values of which are chosen by the user, are classified as bursts. For our purposes, the values of the parameters were those specified in the *NeuroExplorer Manual* (Nex Technologies 2014); see Table 1.

When applied to data sets consisting of multiple spike trains, for example, multiple channels from a single MEA recording, most burst detectors analyze each spike train individually, calculating any associated parameter values, e.g., maxISI, separately for each spike train. The exceptions to this are the MaxInterval (MI) method, which uses the same fixed parameters to detect bursts on all electrodes, and the robust Gaussian surprise (RGS) method, which combines the ISIs from all channels and uses this pooled data set to determine the characteristics of the “central distribution” and find the initial bursting periods on each electrode.

### Analysis of Synthetic Data

The performance of each method was evaluated against a list of properties that we deemed desirable in a burst detector, shown in Table 2. For *properties D1–D3*, performance was based on the details of the method's implementation, while for the remaining properties (*D4–D11*), testing on simulated data was performed. Simulated data were used for this purpose because they allowed us to generate spike trains with specific properties of interest. By explicitly generating periods of bursting activity in these spike trains, we were also able to compare the results of each burst detector with the “ground truth” bursting behavior. Simulated spike trains were produced with the models outlined below, with the parameter values specified in Table 3.

**Table 2.** Desirable properties for a burst detector

*D1*	Deterministic: The method should detect the same bursts over repeated runs on the same data, to ensure consistency and reproducibility of results.
*D2*	No assumption of spike train distribution: The method should not assume that ISIs follow a standard statistical distribution, to ensure wide applicability to a variety of spike trains.
*D3*	Number of parameters: The method should have few parameters, to reduce the variability inherently introduced through parameter choice.
*D4*	Computational time: The method should run in a reasonable amount of time on standard personal computers.
*D5*	Nonbursting trains: The method should detect few spikes as being within bursts in spike trains containing no obvious bursting behavior.
*D6*	Nonstationary trains: The method should detect few spikes as being within bursts in spike trains with nonstationary firing rates that contain no obvious bursting behavior.
*D7*	Regular short bursts: The method should detect a high proportion of spikes in bursts in spike trains containing short, well-separated bursts.
*D8*	Nonstationary bursts: The method should detect a high proportion of spikes in bursts in spike trains containing bursts with variable durations and numbers of spikes per burst.
*D9*	Regular long bursts: The method should detect a high proportion of spikes in bursts and an accurate number of bursts in spike trains containing long bursts with low within-burst firing rates.
*D10*	High frequency bursts: The method should detect a high proportion of spikes in bursts and an accurate number of bursts in spike trains containing a large number of short bursts.
*D11*	Noisy train: The method should classify a high number of within-burst spikes as bursting and a low number of interburst spikes as bursting in spike trains containing both bursts and noise spikes.

**Table 3.** Models and parameter values used to generate synthetic spike trains for each desirable property

Spiking Model	Property	Parameters	Mean % Spikes in Bursts
100 Poisson spiking	Computational time (*D4*)	λ = 1 Hz	0
50 Poisson spiking	Nonbursting (*D5*)	λ = 0.5 Hz, *N* = 50	0
50 Gamma-distributed ISIs		α = 1, β = 0.5, *N* = 50	
100 Inhomogeneous Poisson	Nonstationary (*D6*)	λ(*t*) = 1 + 1/300 *t*	0
100 Poisson bursting	Short bursts (*D7*)	λ = 0.2 Hz, *n* = 5, *r* = 0.3 s	100
100 Poisson bursting	Nonstationary bursts (*D8*)	λ = 0.3 Hz, *n* ∼ U(5, 18),	100
		*r* ∼ U(0.3, 3) s	
100 Poisson bursting	Long bursts (*D9*)	λ = 0.1 Hz, *n* = 18, *r* = 3 s	100
100 Poisson bursting	High frequency (*D10*)	λ = 1 Hz, *n* = 10, *r* = 0.5 s	100
100 Poisson bursting with gamma-distributed noise ISIs	Noisy train (*D11*)	λ = 0.5 Hz, *n* = 8, *r* = 0.8 s	91
		α = 1, β = 0.5

Each spike train was 300 s in duration, and the number, *N*, of simulated trains was 100, unless otherwise stated. α and β represent the shape and inverse scale parameters of the gamma distribution, respectively.

#### Poisson and gamma distributions.

Two types of nonbursting spike trains were simulated, one with Poisson-distributed ISIs and the other with gamma-distributed ISIs. The smallest 10th percentile of ISIs were removed from each spike train by omitting the corresponding spikes, to eliminate any burstlike behavior arising randomly in the simulated data.

#### Inhomogeneous Poisson distribution.

Spike trains with nonstationary firing rates and no bursts were simulated with a Poisson process with nonhomogeneous intensity, λ(*t*). To eliminate any possible bursting behavior, spikes corresponding to the smallest 10th percentile of ISIs were removed from each spike train.

#### Poisson bursting.

Bursting spike trains were simulated with the Poisson bursting model. The location of the center of each burst on a spike train was modeled with a Poisson process with a fixed rate, λ. The number of spikes in each of the bursts was drawn from a Poisson distribution with mean *n*. The position of the spikes in each burst relative to the burst center was drawn from a uniform distribution with range *r* and mean 0. Where two bursts overlapped, only the first was kept.

To simulate spike trains with nonstationarity in their bursting properties, the values of *n* and *r* were drawn randomly from a uniform distribution for each burst, rather than being held as fixed values. Only the resulting bursts with within-burst firing rate above 5 Hz were retained.

To simulate noise in bursting spike trains, noise spikes were modeled with gamma-distributed ISIs with the smallest 10th percentile of ISIs removed. These noise spikes were added to the Poisson bursting spike train, and any noise spikes within 0.5 s of the limits of each burst were removed, to prevent any overlap between burst and noise spikes.

**Fig. 1.**
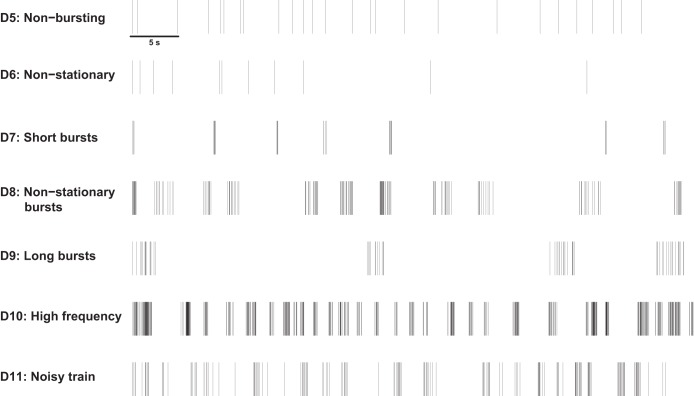
One-minute examples of simulated spike trains for evaluating desirable properties *D5–D11*.

For each desirable property, 100 spike trains of duration 300 s were simulated and analyzed using each of the burst detectors detailed above. Examples of the simulated spike trains used for evaluating each desirable property are shown in Fig. 1. A comparison of the “ground truth” bursting activity and the results from each burst detector was then performed. For spike trains containing both bursts and noise spikes, this involved examining the fraction of true positive spikes, defined as the proportion of within-burst spikes correctly identified as being in bursts, and the fraction of false positive spikes, defined as the fraction of all noise spikes erroneously identified as being within bursts, found by each burst detector.

### Analysis of Mouse RGC Data

**Fig. 2.**
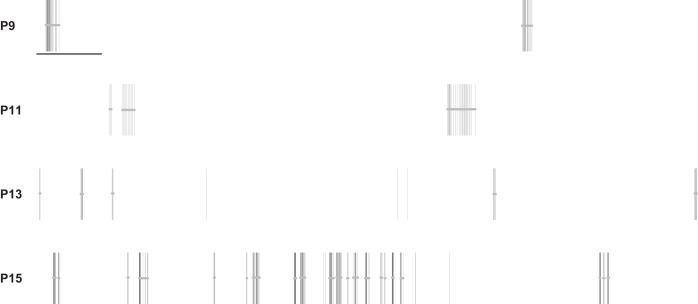
Examples of 1-min spike trains from recordings of mouse retinal ganglion cells at each postnatal day. Horizontal bars represent bursts annotated by a human observer. Scale bar represents 5 s of activity.

MEA recordings of mouse RGCs from Demas et al. (2003) were reanalyzed with the burst detectors in our study. These MEA recordings are available from Eglen et al. (2014). Four 1-h-long recordings of control mouse retina at postnatal day (P)9, P11, P13, and P15 were chosen for reanalysis. For spike trains from five randomly chosen electrodes from each recording, bursts were annotated by visual inspection by one of the authors (E. Cotterill). Figure 2 shows examples of annotated bursts for spike trains at each age. As MEA recordings do not provide the “ground truth” location of bursts, these visually identified bursts were taken as a proxy for “ground truth” bursts and used to compare the results from each burst detector against. Comparison to visually identified bursts has been used previously to assess burst detection techniques (Chen et al. 2009; Gourévitch and Eggermont 2007; Pasquale et al. 2010). For each burst detector in our study, bursts were detected on the annotated spike trains with a variety of input parameters and the sensitivity and specificity of each method examined.

To assess their robustness, we chose a key parameter to vary for each burst detector. The parameter that was varied to examine the sensitivity and specificity of the HSMM and surprise-based methods was the probability cutoff, while for the ISI rank threshold (IRT) method, the spike count cutoff, θ_*C*_, was altered. For the logISI method, the limit on the maximum allowed ISI cutoff value was varied from its initial value of 100 ms. For the MI method, most parameter values shown in Table 1 were maintained, excluding the maximum beginning and end ISIs, which were varied so that the maximum end ISI was always 0.130 s greater than the maximum beginning ISI. Finally, for the CMA method, for which there were no obvious parameters to vary, only a single value for sensitivity and specificity was found.

Receiver operating characteristic (ROC) curves were produced that plotted 1 − specificity vs. sensitivity for various parameter values. Sensitivity was defined as the number of spikes correctly detected as being within bursts, as a fraction of the total number of spikes in the visually annotated bursts. The value of 1 − specificity, or the false positive rate, was the number of spikes that were falsely detected as being within bursts, as a fraction of the total number of spikes that were not a part of the “ground truth” bursts.

### Experimental Details for hiPSC-Derived Neural Network Recordings

Neuronal networks were grown from late-stage neuronal precursors differentiated from hiPSCs (Axol Bioscience, Cambridge, UK). hiPSCs were generated by reprogramming of embryonic cord blood cells and then differentiated to the neural lineage with protocols based on those in Shi et al. (2012). All of the recordings (447 recordings from 73 MEA platings on 11 plating dates, 4 thawed vials) were obtained with a single line and neural induction (AX0015; https://www.axolbio.com/page/neural-stem-cells-cerebral-cortex).

Late-stage neuronal precursors (1 × 10^6^) were thawed and expanded by growing on six-well tissue culture plates coated with polyornithine and laminin (2 × 10^5^ cells/well) in Neural Maintenance Medium (NM), supplemented with 10 μM Y-27632 (rho-associated protein kinase inhibitor) for the first 24 h. After 4–5 days cells were dissociated with Accutase, centrifuged, resuspended in NM, and either plated to MEAs (2 × 10^4^−1 × 10^5^) or expanded further on six-well plates as above. MEAs (60MEA200/30-Ti; Multi Channel Systems, Reutlingen, Germany) were coated with polylysine followed by laminin as described previously (Charlesworth et al. 2016).

hiPSC-derived neuronal network (hiPSN)-MEA cultures were maintained in NM medium under zero-evaporation lids (Potter and DeMarse 2001) housed in tissue culture incubators maintained humidified at 37°C and 5% CO_2_-10% O_2_-85% N_2_. Medium was completely exchanged after 24 h to remove Y-27632. Thereafter, MEA cultures were fed by exchanging 40–50% medium with fresh NM three times per week. NM composition was a 1:1 mixture of N2 and B27 supplemented media (N2 medium: DMEM-F-12 + N2 supplement and 5 μg/ml insulin, 1 mM l-glutamine, 100 μM nonessential amino acids; B27 medium: Neurobasal Medium + B27 supplement and 0.5 mM l-glutamine).

Recordings (300 s) of spontaneous extracellular neuronal activity in hiPSN-MEA cultures were made weekly with an MEA system supplied by Multi Channel Systems (MEA 1060INV, with 60MEA200/30-Ti arrays; titanium nitride electrodes, 30-μm diameter, 200-μm spacing, internal reference electrode). The signal was sampled at 25 kHz and stored with a 64-channel data acquisition board (MC Card; Multi Channel Systems) and the acquisition software MCRack (Multi Channel Systems). Action potentials were detected by crossing of a threshold set to a level of 6 standard deviations from the baseline noise level. Record samples (1 ms before and 2 ms after crossing of threshold) confirmed the characteristic action potential waveform. Action potential timestamps were extracted to text file with batch scripts written for NeuroExplorer (Nex Technologies, Littleton, MA). Recordings made at dates above 16 weeks after plating (WAP) were excluded from the analysis because of the small number of data points, resulting in 424 recordings being analyzed. All experiments using human stem cells were vetted and approved by the Steering Committee for the UK Stem Cell Bank and for the Import of Stem Cell Lines in 2012. All procedures were compliant with the UK Code of Practice for the Use of Human Stem Cell Lines.

## RESULTS

### Desirable Properties for a Burst Detector

To evaluate the performance of each burst detector, the methods were assessed against 11 desirable properties, listed in Table 2. The optimal burst detector would ideally possess all of these desirable properties. For binary properties, *D1–D4*, each method was judged to either possess the property or not, while for properties *D5–D11*, the performance of each method was ranked against the other methods, based on the median and variance of its performance at analyzing 100 synthetic spike trains.

The first desirable property of a burst detector was that it was deterministic (*D1*), as this ensures reproducibility and removes the need to find a “consensus” set of bursts across repeated trials. The only nondeterministic burst detector was the HSMM method, because of its use of MCMC methods. The bursts detected by this method varied considerably between trials. For example, when used repeatedly to analyze one simulated 300-s Poisson bursting spike train with burst frequency of 0.2 Hz, the HSMM method identified 51 ± 9.75 bursts (mean ± SD) over 100 trials.

Another desirable property for the burst detectors was that they did not assume that ISIs follow a specific statistical distribution (*D2*). There is no consensus on which type of statistical distribution best represents underlying spike train activity, and any assumption that this activity follows a fixed statistical distribution restricts the applicability of a method to a narrow range of spike trains. Most methods do not assume a fixed statistical distribution for the underlying spike train, excluding the PS, RGS, and HSMM methods, which assume that ISIs can be modeled with a Poisson process, Gaussian distribution, and gamma distributions, respectively. However, the PS and RGS methods detect bursts as periods of deviation from these underlying firing rate distributions. These methods thus remain somewhat robust when the distribution assumptions are not met, as “surprising” sequences of spikes as measured by one distribution will generally also correspond to high-surprise values from other distributions commonly used to model spike trains (Legéndy and Salcman 1985).

A common issue that arises when applying burst detection techniques to large sets of spike trains that have high variability in their statistical properties, such as those from MEA recordings of human neuronal networks, is how to accurately choose the parameters for burst detection. This is further confounded when burst detectors are used to analyze MEA recordings spanning a large range of developmental ages or differing experimental conditions. Thus, ideally, a burst detector should have few parameters (*D3*), to minimize the impact of how parameter values affect the resultant detected bursts. Most methods in our study only required one or two parameters. The MI method, however, required five parameters to implement burst detection. The HSMM method also required a large number (*n* = 23) of parameters; however, many of these are initial values that are later optimized by the algorithm, and can be left set to the values suggested by the original authors with little impact on the effectiveness of the method.

With the increasing prevalence of high-density MEAs that contain up to several thousand electrodes (Maccione et al. 2014), as well as the use of multiwell MEAs in applications such as high-throughput neurotoxicology screening (Nicolas et al. 2014; Valdivia et al. 2014) and drug safety testing (Gilchrist et al. 2015), the computational complexity of each method must also be considered. To assess computational time (*D4*), each method was used to analyze 100 simulated spike trains of 5-min duration with average firing rate of 1 Hz. Most methods required on average only a fraction of a second to analyze each spike train with a standard personal computer. The exception to this was the HSMM method, which had an average computational time >20 times greater than any other method.

**Fig. 3.**
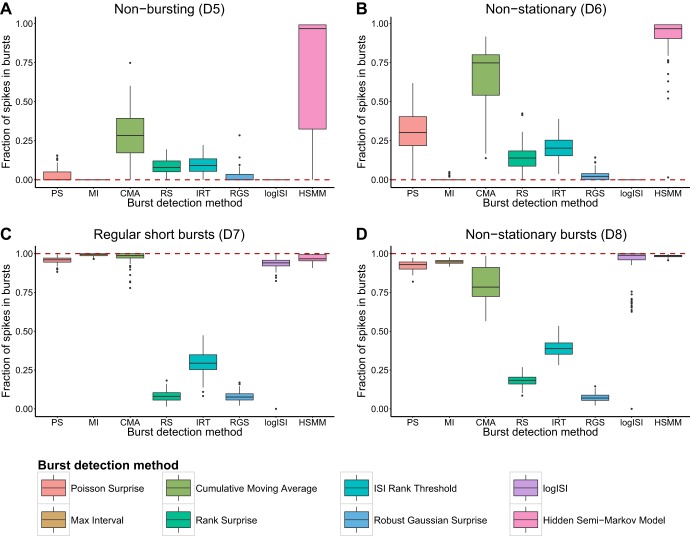
Fraction of spikes in bursts found by each burst detector in 100 synthetic trains with no bursting (*D5*, *A*), no bursting and nonstationary firing rate (*D6*, *B*), short regular bursts (*D7*, *C*), and bursts with nonstationary burst lengths and durations (*D8*, *D*). Dashed line shows desired result from an ideal burst detector; methods close to this line are deemed to work well. In each box-and-whisker plot, boxes show the median ± interquartile range (IQR), and whiskers extend to median ± 1.5 × IQR. Outliers are represented as points.

A common feature seen in MEA recordings of human neuronal networks is many electrodes that record sparse or no bursting behavior. An ideal burst detector would find no or very little bursting activity in these spike trains. Most burst detectors performed reasonably well at detecting a low amount of bursting activity in spike trains simulated to exhibit an absence of bursting behavior (*D5*). The major exception to this was the HSMM method, which had a tendency to significantly overestimate bursting behavior in these spike trains (Fig. 3*A*).

When a nonstationary firing rate was incorporated into nonbursting spike trains (*D6*), the number of erroneous bursts detected by most methods increased (Fig. 3*B*). The PS and CMA methods, in particular, showed a significant increase in the amount of bursting activity detected in nonstationary spike trains, compared with those with a static mean firing rate. These methods tend to detect periods of “unusual” activity as bursts and thus showed a tendency to detect bursts in the regions of relatively high firing rate in these spike trains.

An ideal burst detector should also detect bursts accurately in spike trains that contain only bursting activity, especially those in which the bursts are regular and well separated (*D7*). Most methods possessed this property and could identify >90% of the spikes within bursts in simulated spike trains containing regular bursting behavior (Fig. 3*C*). The exceptions to this were the RS, IRT, and RGS methods, which consistently detected less than half of the bursts in these synthetic spike trains. This result is unsurprising, since these three methods use thresholds that impose a limit on the maximum proportion of ISIs in a spike train that can be classified as being within bursts.

We also analyzed the performance of each burst detector on simulated spike trains with less standard bursting behavior. This included spike trains containing nonstationary bursting activity (*D8*), in the form of bursts with variable lengths and durations. The logISI, HSMM, PS, and MI methods correctly identified most spikes in bursts in these spike trains (Fig. 3*D*). The fraction of bursting spikes detected by the CMA method varied considerably across the 100 simulated spike trains, and it usually correctly identified a significantly lower proportion of within-burst spikes in these spike trains, compared with those containing regular bursting activity. The RS, IRT, and RGS methods continued to detect only a small proportion of the bursting activity.

**Fig. 4.**
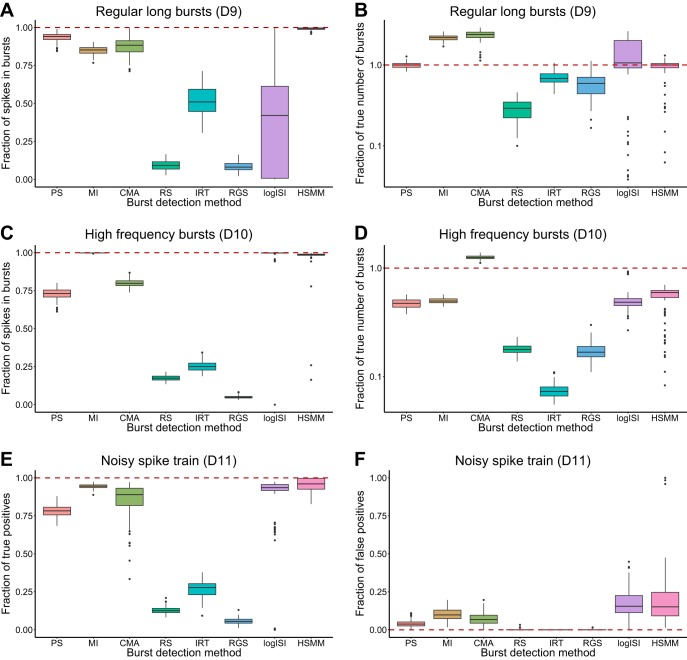
Results of each burst detector at analyzing 100 synthetic spike trains: fraction of spikes in bursts (*A*) and fraction of true number of bursts (*B*) in spike trains with regular long bursts (*D9*); fraction of spikes in bursts (*C*) and fraction of true number of bursts (*D*) in spike trains with high-frequency bursting (*D10*); fraction of true positive (*E*) and fraction of false positive (*F*) spikes in bursts in spike trains containing both bursting and noise (*D11*). Values calculated as outlined in materials and methods. Box plots and dashed line as in Fig. 3. *B* and *D* are presented on a log scale.

We also examined the performance of each burst detector on spike trains containing bursts with long durations and relatively low within-burst firing rates (*D9*). For these spike trains, only the PS and HSMM methods gave reasonably accurate results for both the fraction of spikes in bursts and the number of bursts in the spike trains (Fig. 4, *A* and *B*). The MI and CMA methods both correctly allocated a large proportion of the spikes as being within bursts but tended to separate the long bursts into shorter, more frequent bursts, while the remaining methods greatly underestimated the prevalence of bursting activity in the simulated data (Fig. 4, *A* and *B*).

Another type of nonstandard bursting activity seen in human network recordings is the presence of short, poorly separated bursts occurring at a high frequency. When used to analyze spike trains with very frequent bursting behavior (*D10*), the MI, logISI, and HSMM methods could correctly identify the majority of spikes as being within bursts but had a tendency to combine the short bursts into a smaller number of bursts with longer durations (Fig. 4, *C* and *D*). The CMA method most accurately detected the large number of bursts in these high-frequency spike trains but tended to underestimate the proportion of spikes in bursts. The RS, IRT, and RGS methods were only able to identify a low fraction of bursting spikes and low number of bursts in these spike trains (Fig. 4, *C* and *D*).

Finally, an ideal burst detector should correctly differentiate between bursting and nonbursting periods in spike trains in which some spiking activity occurs outside of bursts (*D11*). By comparing each method's output to the ground truth bursting behavior in simulated spike trains containing both bursts and noise, we examined the fraction of correctly identified within-burst spikes, as well as the fraction of noise spikes erroneously detected as being within bursts. The MI, CMA, logISI, and HSMM methods displayed reasonably high true positive rates for identifying bursting spikes; however, of these, the logISI and HSMM methods tended to classify a higher proportion of noise spikes as being within bursts (Fig. 4, *E* and *F*). The RS, IRT, and RGS methods exhibited very low false positive rates, but this came at the expense of quite low true positive rates, giving them low overall recall. The performance of the PS method was between these two extremes, with both lower true positive and false positive rates than the highest-performing burst detectors.

Table 4 and Table 5 summarize the performance of each burst detector across all desirable properties. The ranking in Table 5 was based on the median and range of the box plots in Fig. 3 and Fig. 4, with methods with similar results given equal rankings. Three methods clearly underperformed based on this ranking, namely, the RS, IRT, and RGS methods. To further assess the performance of the burst detectors, they were each used to analyze bursting activity in experimental recordings from mouse RGCs.

**Table 4.** Summary of performance of each method on desirable properties D1–D4

	PS	MI	CMA	RS	IRT	RGS	LogISI	HSMM
*D1*: deterministic	✓	✓	✓	✓	✓	✓	✓	x
*D2*: distribution assumption	x	✓	✓	✓	✓	x	✓	x
*D3*: number of parameters	✓	x	✓	✓	✓	✓	✓	x
*D4*: computational time	✓	✓	✓	✓	✓	✓	✓	x

**Table 5.** Relative rank of performance of each method on desirable properties D5–D11

	PS	MI	CMA	RS	IRT	RGS	LogISI	HSMM
*D5*: nonbursting	4	1	7	5	6	3	1	8
*D6*: nonstationary	6	2	7	4	5	3	1	8
*D7*: regular bursting	4	1	2	7	6	7	5	3
*D8*: nonstationary bursts	4	3	5	7	6	8	2	1
*D9*: long bursts	2	4	3	8	5	7	6	1
*D10*: high frequency	5	1	4	7	6	8	2	3
*D11*: noisy bursts	5	1	2	7	6	8	4	2
Total (relative rank)	30(4)	13 (1)	30 (4)	45 (8)	40 (6)	44 (7)	21 (2)	26 (3)

1 = best, 8 = worst.

### Preliminary Analysis of Mouse RGC Data

**Fig. 5.**
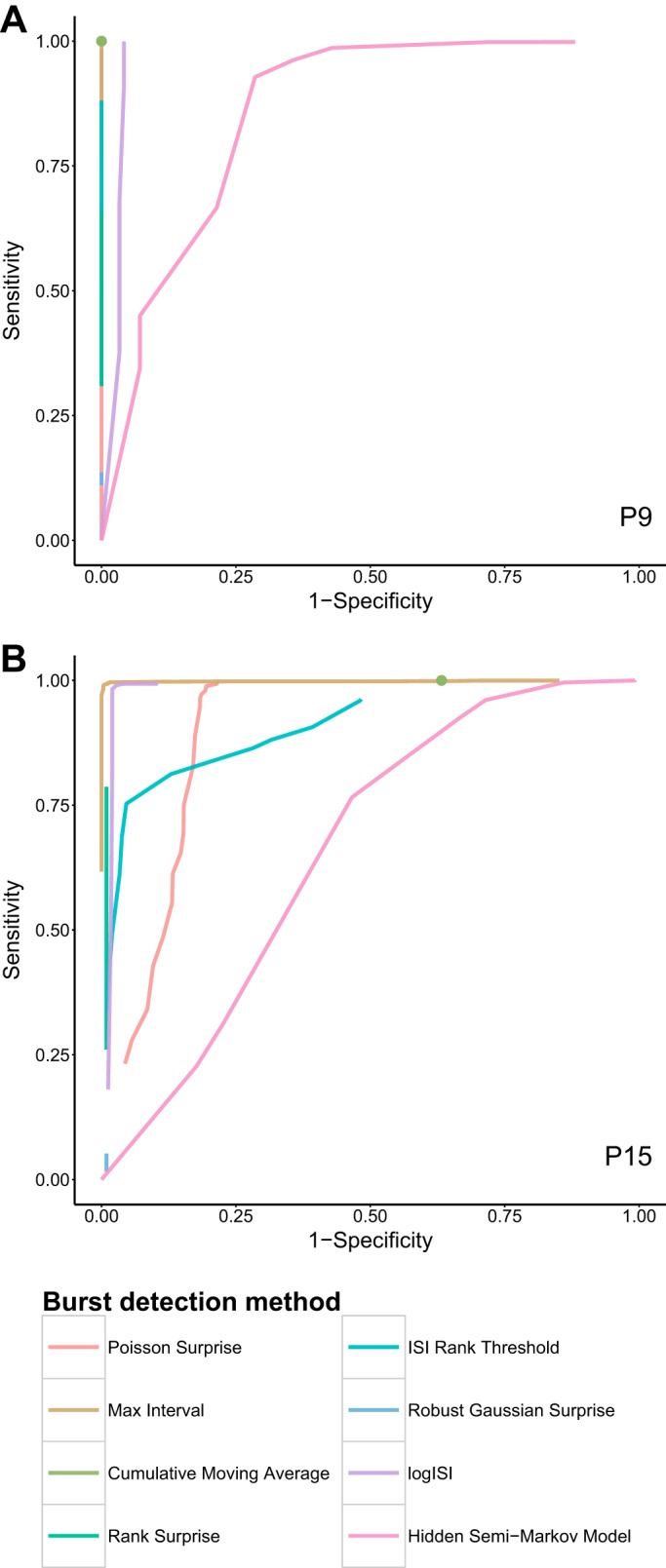
ROC curves showing the fraction of true positive (sensitivity) and false positive (1 − specificity) spikes identified as being within bursts for a variety of input parameter values, for recordings of mouse retinal ganglion cells at P9 (*A*) and P15 (*B*). The ground truth bursts for hour-long recordings from 5 randomly selected electrodes at each age were determined by visual inspection (examples in Fig. 2), and the mean performance of each burst detector over the 5 electrodes is shown. Some curves do not span the entire range because of innate restrictions on the maximum proportion of spikes that can be allocated to bursts by each method. Green dot represents the single specificity and sensitivity value found by the CMA method, which has no obvious parameter to vary.

MEA recordings of mouse RGCs from Demas et al. (2003), a study that examined the developmental changes in spontaneous retinal activity in normal and dark-reared mice, were reanalyzed with our burst detectors. The sensitivity and specificity of each method at a range of parameter values was calculated, and averaged across the five annotated spike trains from each recording, to produce the ROC curves in Fig. 5. The ROC curves for P11 and P13 are omitted, as they resemble the results at P9. Because of innate restrictions on how bursts are defined by each method, for example, that bursts must contain a minimum of three spikes, many burst detectors did not allocate either no spikes or 100% of spikes as being within bursts for any choice of parameter values, and thus the ROC curves do not span the entire range of sensitivity and specificity values. The methods were thus assessed by their minimum distance from the point of perfect classification at (0,1), rather than the area under the ROC curve.

The MI method exhibited strong performance across all ages and reached very high levels of sensitivity and specificity for a specific choice of parameter values at each age. The logISI, PS, and CMA methods also had promising performance across most ages; however, at P15 the PS and CMA methods exhibited higher false positive rates (Fig. 5*B*). The results from the RGS method did not vary significantly as its parameter value was changed, and it was unable to reach high levels of sensitivity for any choice of parameter values. The sensitivity and specificity of the RS and IRT methods, on the other hand, spanned a range of values; however, these methods did not reach the levels of sensitivity of the other methods. The HSMM method reached high sensitivity levels, but only at the cost of low specificity, and generally performed poorly across all ages.

### Evaluation of Methods

As shown by the assessment of the burst detectors against the desirable properties, the RS, IRT, and RGS methods underperformed compared with the other methods. This was reinforced through their performance when compared with visually annotated bursts in mouse RGC recordings, where they did not reach the levels of sensitivity of the other methods in our study. These three methods were thus eliminated from further analysis. The HSMM method had average performance on simulated data (*D5–D11*); however, its complex implementation meant that it was the lowest-performing method on properties *D1–D4*. The high false positive rate of the HSMM method across all ages when analyzing experimental data cemented our decision to exclude this method from further consideration.

### Further Analysis of Mouse RGC Data

The remaining four methods (PS, MI, CMA, and logISI) were used to analyze the complete set of spike trains from each of the four control mouse RGC recordings from Demas et al. (2003). In this case, the parameters used for the analysis were based on those that resulted in the best performance in the ROC curves, as measured by the distance of the curve from the point of perfect classification in the top left corner. In the original report, rather than explicitly identifying the location of bursts, Demas et al. (2003) used the autocorrelation of each spike train to determine the average burst duration at each age. By explicitly identifying bursts using our four burst detectors, we were able to provide a more detailed description of the bursting activity and compare this with the authors' original results.

**Fig. 6.**
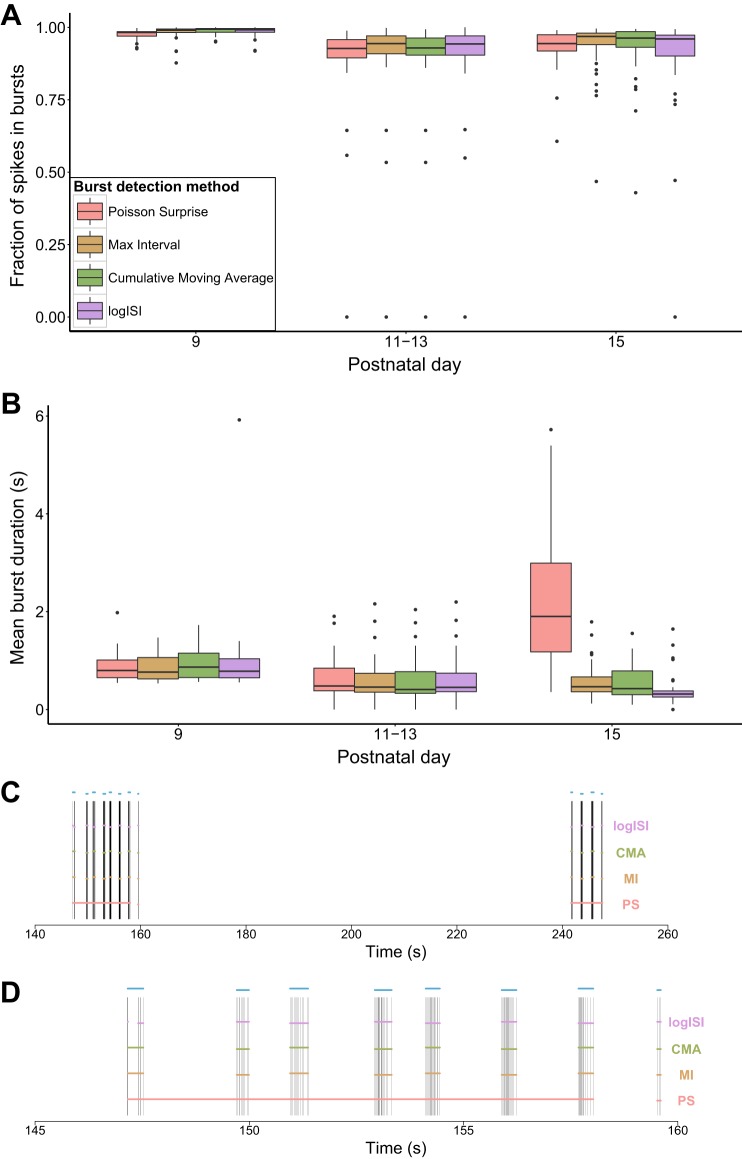
Detailed analysis of mouse retinal ganglion cell recordings. *A* and *B*: fraction of spikes in bursts (*A*) and mean burst duration (*B*) found by each burst detector. Each electrode was counted as 1 data point in the box plots. Key in *A* applies to both *A* and *B*. *C* and *D*: bursts detected by each burst detector over a 120-s sample of a P15 spike train (*C*) and a 15-s sample showing the first bursting episode from the same spike train (*D*). Horizontal bars in *C* and *D* denote the bursts detected by each method. Blue bars above the spike train represent the bursts annotated by a human observer.

The four burst detectors were generally in agreement about the proportion of spikes in bursts across all ages and showed a decrease in the fraction of spikes in bursts with increasing developmental age (Fig. 6*A*). This concurs with the analysis of the original authors, who found that only very few spikes occurred outside of bursts at early ages, while by P15 many cells were active outside of bursts (Demas et al. 2003).

In terms of burst duration, all four methods showed very similar results over P9–P13, which resemble the values found by the autocorrelogram method. However, at P15 there was a significant deviation between the results of the PS method and the other burst detectors. The MI, CMA, and logISI methods followed the trend of decreasing burst duration with age, as in the original analysis, while the PS method detected a significant increase in burst duration at P15. This can be attributed to the fact that at P15 many spike trains exhibited regular “bursting episodes,” periods of high activity generally spanning 10–20 s that consisted of a series of shorter bursts. The PS method had a tendency to classify these bursting episodes as one long burst, while the other methods generally broke up these periods into several shorter bursts, as shown in the example spike train in Fig. 6*D*.

### Analysis of hiPSC-Derived Neuronal Network Recordings

To further assess these methods, 424 MEA recordings from 73 hiPSC-derived neuronal networks recorded at regular intervals from 2 to 16 WAP were analyzed with the four best burst detectors. The MI, PS, and logISI parameters used for this analysis were chosen by inspection as those that most accurately detected bursts on five randomly chosen spike trains with mean firing rate close to 1 Hz, which is the minimum firing rate at which regular bursting activity tends to arise. For the CMA method, the scale for α_1_ values from Kapucu et al. (2012) was used, and the authors' suggestions for post hoc screening were employed, with any spike trains that were found to have average burst duration > 5 s or an average burst length > 50 spikes per burst declared as nonbursting. The resultant parameters used to implement each method are shown in Table 6.

**Table 6.** Parameter values used for burst detection on human induced pluripotent stem cell-derived neuronal networks

Method	Parameter	Value
Poisson surprise	Minimum surprise value	−log(0.0025) ≈ 6
MaxInterval	Maximum beginning ISI	0.2 s
	Maximum end ISI	0.3 s
	Minimum interburst interval	0.2 s
	Minimum burst duration	0.01 s
	Minimum spikes in a burst	3
Cumulative moving average	α_1_	1.0 if skew < 1
		0.7 if 1 ≤ skew <4
		0.5 if 4 ≤ skew <9
		0.3 if 9 ≤ skew
	Maximum mean burst duration	5 s
	Maximum mean spikes per burst	50
LogISI	Maximum cutoff value	150 ms

**Fig. 7.**
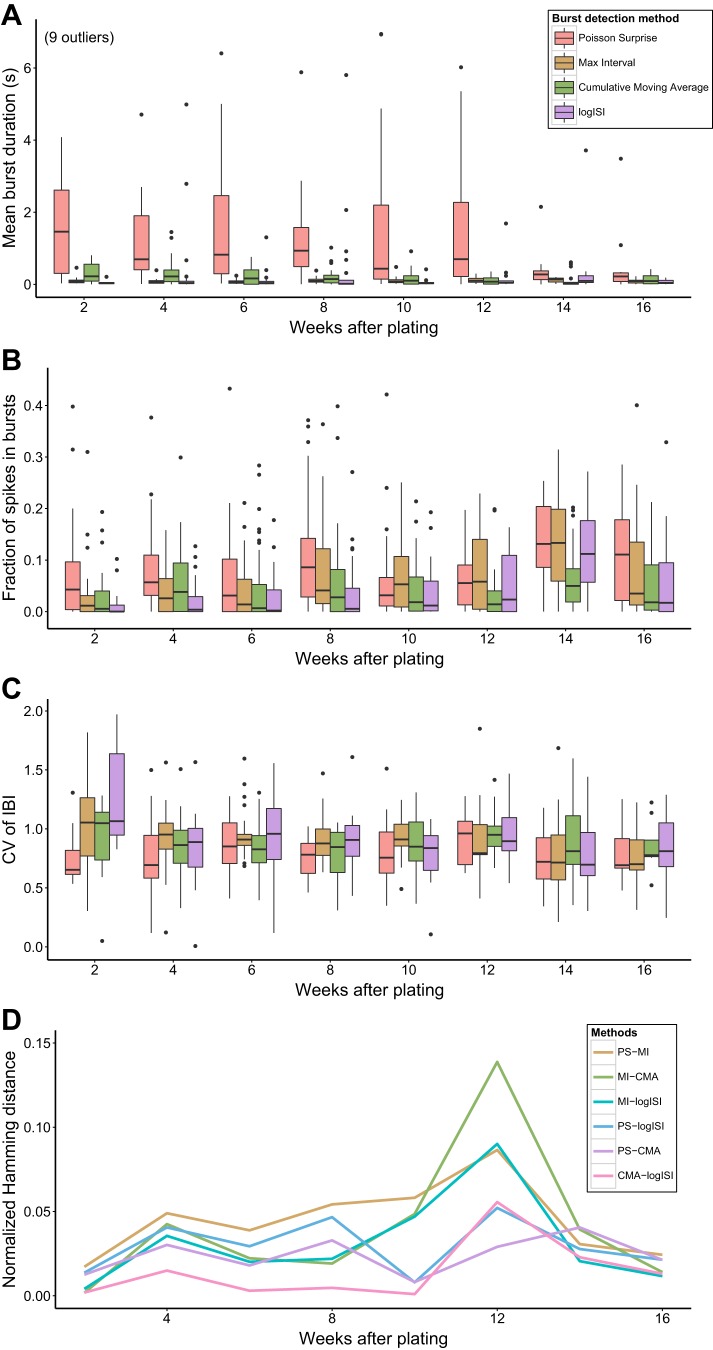
Analysis of recordings of networks of human induced pluripotent stem cell-derived neurons. *A*: mean burst duration. *B*: fraction of spikes in bursts. *C*: coefficient of variation of interburst intervals (CV of IBI). Each data point in the box plots is the mean value across all electrodes from 1 recording. *D*: median normalized Hamming distance between each pairwise combination of burst detection methods at each week after plating.

Although there were some differences in the absolute level of bursting activity detected by the different analysis methods, the results from most methods suggested a general trend of “ramping up” of bursting, in terms of fraction of spikes in bursts, with increasing developmental age (Fig. 7*B*). In general, however, the prevalence of bursting activity in these human network recordings tended to be significantly lower than that commonly seen in recordings of rat and mouse hippocampal or cortical networks (Charlesworth et al. 2015; Chiappalone et al. 2005). The results also suggest that the prevalence of bursting activity in these networks may decrease with age after reaching a peak around 14 WAP (Fig. 7*B*). This would be consistent with previous studies using calcium imaging of human pluripotent stem cell-derived neuronal networks, which found that bursting activity decreases at later stages of development, when it is replaced by more complex firing patterns (Kirwan et al. 2015). However, additional recordings at later time points would be required to confirm this trend in our data.

Unlike some previous studies of rodent neuronal networks (Charlesworth et al. 2015; Demas et al. 2003), there was no obvious relationship between burst duration and culture age in our hiPSC-derived network recordings, with bursts remaining short over the entire developmental period. Similarly, the degree of regularity of the bursting activity, captured by the coefficient of variation of interburst intervals (CV of IBI), did not appear to change significantly with increasing developmental age (Fig. 7, *A* and *C*).

To quantify the differences between the bursts found by each burst detector in these recordings, we converted each spike train to a time series by dividing the 300-s recording period into 50-ms bins. A binary vector was then found for each burst detector, which took a value of 1 if the spike train was found to be in a bursting state during that time bin or 0 otherwise. The Hamming distance, which represents the number of points at which two binary strings differ, was calculated between each pair of methods for every spike train on which bursting activity was detected by all four methods and normalized to represent the fraction of time bins in which the results from each pair of burst detectors differed.

**Fig. 8.**
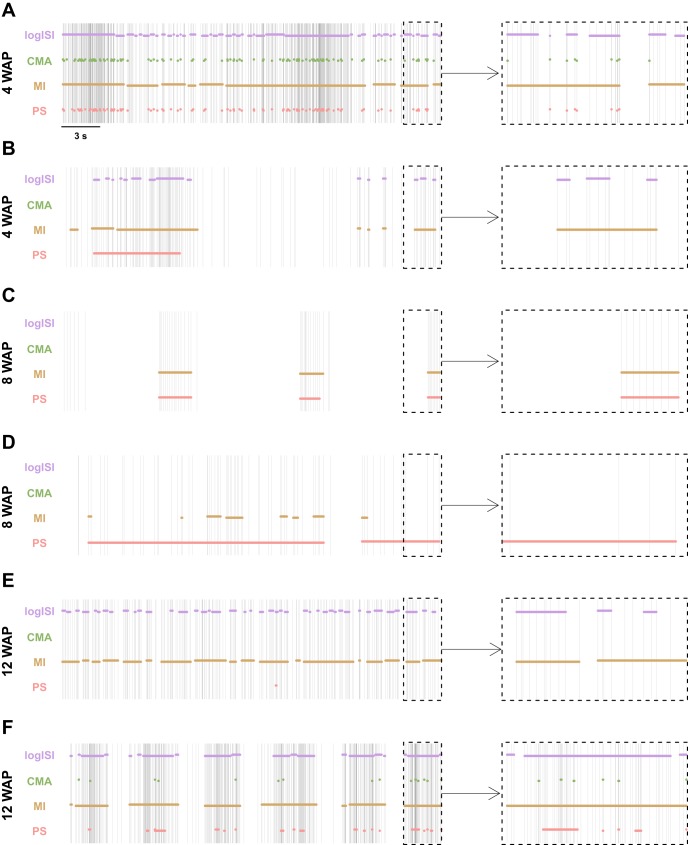
Results of the 4 burst detectors applied to samples of human induced pluripotent stem cell-derived neuronal network recordings at 4 weeks after plating (WAP) (*A* and *B*), 8 WAP (*C* and *D*), and 12 WAP (*E* and *F*). *Left*: spike trains show 30 s of activity. *Right*: enlarged version of the last 3 s of this activity. Horizontal bars denote the bursts detected by each method.

Figure 7*D* shows that the median Hamming distance between most of the methods was <5% at most WAPs. At 12 WAP, however, there was a peak in Hamming distances, in particular those measuring the difference between the bursts detected by the MI method and other burst detectors. At 12 WAP, the recordings on average exhibited a higher mean firing rate and lower variability of ISIs compared with recordings at other time points, with many electrodes recording tonic spiking or bursting activity at a high frequency (e.g., Fig. 8, *E* and *F*). As the MI method detects bursts based on the absolute length of ISIs, this method had a tendency to find a large proportion of bursting activity in these high-frequency spike trains, while the other methods, which detect bursts as periods of high firing rate relative to the background activity, generally detected a much lower proportion of bursting activity in these spike trains. Altering the MI method parameters at this WAP, to reduce the maximum allowed beginning and end ISIs in a burst, could bring its results more in line with those of the other burst detectors.

Visual inspection of spike trains at other WAPs was also performed to gather insight on the differences between the bursts detected by each method at these ages (Fig. 8). In several examples, the CMA method failed to detect numerous periods that visual inspection and the other burst detectors generally classified as bursting (Fig. 8, *B* and *C*). This may account for the lower proportion of spikes in bursts found by this method, compared with the other burst detectors (Fig. 7*B*). The logISI method also detected low proportions of spikes in bursts across many WAPs (Fig. 7*B*). This may explain the generally low Hamming distances between the CMA and logISI methods (Fig. 7*D*). Additionally, the PS method tended to combine bursts that other methods detected as separate bursts and extend bursts to incorporate additional spikes that visual inspection would suggest should not be included in bursts, accounting for the longer burst durations found by this method (Fig. 7*A*). Although no method agreed perfectly with how we would assign bursts in these recordings of hiPSC-derived neuronal networks, when a large subset of spike trains were visually examined, of the four methods examined here on average the MI method corresponded most closely to how we would annotate bursts visually.

## DISCUSSION

Despite the important role of accurate burst detection in analyzing neuronal network activity in a variety of contexts, a consistently widely used method for burst analysis is yet to be adopted. By examining the performance of eight burst detectors at analyzing both synthetic and experimental data, we found that a number of existing methods perform poorly at identifying bursts in spike trains with a variety of properties. We identified four burst detectors that outperformed compared with other existing methods and used these to analyze bursting activity in recordings of hiPSC-derived neuronal networks over several months of development.

We have shown that a number of burst detectors that were developed based on recordings from single experimental conditions do not necessarily generalize to use on other types of spike trains. For example, the RGS method, which was originally developed to analyze dopaminergic neurons, could not detect the majority of bursts in simulated spike trains and also performed poorly at analyzing experimental recordings from mouse RGCs, even when its probabilistic threshold parameter was varied over a large range. Other studies have also found issues using the RGS method to analyze changes in bursting behavior under different drug effects (Eisenman et al. 2015).

The IRT method also performed poorly at detecting bursts in a range of different spike trains. Unlike the other methods included in our study, this method was not published in a methods paper but rather was a heuristic method designed for the analysis of a specific data set that was not spike sorted (Hennig et al. 2011), so its lack of adaptability is not surprising.

We have also shown that the complexity of a burst detector does not necessarily correlate with its effectiveness. The most complex method in our study, the HSMM method, often performed only equally well or worse than simpler methods, particularly in nonbursting conditions. Furthermore, the high computational time and nondeterministic nature of this method severely limit its ability to be scaled up for use in high-throughput analysis of MEAs, which is becoming increasingly prevalent in applications such as large-scale neurotoxicity testing (Nicolas et al. 2014).

The performance of other methods was hindered by their underlying assumptions, such as the RS method, which has the tendency to assign approximately the same proportion of spikes as being within bursts in each spike train, regardless of how spikes are distributed. This meant that the RS method tended to both overestimate bursting activity in nonbursting trains and underestimate bursting in spike trains in which most spikes occurred within bursts, making it unsuitable for analyzing MEA recordings in which the level of bursting activity does not remain consistent across all electrodes.

The CMA method, which was designed for the purpose of analyzing recordings from developing human neuronal networks, was a promising candidate in our analysis. The major limitation of this method was its tendency to erroneously detect a large amount of bursting activity in spike trains containing no or sparse bursting activity, in particular those with nonstationary firing rates. The authors' suggestion for post hoc screening can address this issue but also leads to underestimation of bursting in some spike trains, as it does not allow for any shorter bursts to be identified in spike trains in which long erroneous bursts were initially detected by the CMA method.

By our analysis, two burst detectors showed the most promise, namely, the MI and logISI methods. These methods possessed the majority of properties we deemed desirable for a burst detector and were generally able to achieve high coherence with visually detected bursts in experimental data when their parameter values were chosen optimally. These methods, however, still had limitations: The MI method requires the choice of a large number of parameters, the correct value of which can be challenging to determine, and the logISI method had a tendency to underestimate burst durations in some cases.

Given that we have found no “perfect” method for burst detection, our advice is to choose a burst detector based on the number of degrees of freedom the user wishes to control. The MI method consistently outperformed throughout our analysis and is our recommendation for a first choice when selecting a burst detector. Although it has a significant number of parameters to be set by the user, unlike methods with probabilistic thresholds, these parameters are easy to interpret and adjust to achieve the desired burst detection results. If appropriate parameters cannot be found for this method, a high-performing alternative is the logISI method, which can be implemented without choosing any input parameters. This method is most effective when there is a clear distinction between the sizes of within-burst and between-burst intervals. In cases when this distinction is not apparent, we recommend the PS and CMA methods as reasonably effective alternatives. Because of their tendency to overestimate burst durations in some circumstances, however, post hoc screening for outliers in terms of burst duration is advisable when using these methods.

The most robust approach to burst detection would be to apply a number of burst detectors to the data of interest and compare the result of each method using summary statistics or measures such as the Hamming distance. If the methods are in agreement, this provides confidence in the conclusions about the nature of bursting activity in the experimental data. Any major discrepancies between the methods can also be used to pinpoint areas where one or more burst detectors may be performing poorly, an issue that can be further investigated through visual inspection of the specific spike trains of interest. In particular, periods in which the bursts found by the MI method deviate greatly from those found by other methods may suggest that the MI method parameters used were suboptimal for the analysis of these spike trains. In general, we found that for spike trains that are easy to annotate with visual inspection, high-performing burst detectors tend to be in close agreement. However, in spike trains for which two humans may not be able to agree on how to appropriately allocate spikes to bursts, it is likely that the methods will also disagree, and discretion is required.

By employing this method of applying a number of burst detection techniques to recordings of networks of hiPSC-derived neurons over a range of developmental ages, we found that bursting arises in a majority of these networks around 8–10 wk after differentiation. This is a time frame similar to the findings from some previous studies of human stem cell-derived neuronal networks (Heikkilä et al. 2009; Kirwan et al. 2015). Additionally, although we observed some increase in bursting activity over development, the rate of this increase was far lower than that which has been commonly seen in developing rodent neuronal networks (Charlesworth et al. 2015; Chiappalone et al. 2005; Wagenaar et al. 2006).

One limitation of our study was the limited number of burst detectors examined. This was a deliberate choice, because of the extensive number of burst detectors available in the literature, which makes an exhaustive analysis of all methods impossible. Instead of providing a brief analysis of all burst detection methods, we restricted the scope of our study in order to provide a thorough assessment of what we saw as the most promising methods of burst detection and to offer implementable recommendations to researchers working in this area. We also provide R code to implement all of the methods examined here (https://github.com/ellesec/burstanalysis).

The results of our study were also influenced by how the “ground truth” bursts were chosen by visual inspection in the experimental RGC recordings, which is necessarily a subjective choice. However, the relatively high degree of coherence between our visually annotated bursts and those identified by a number of burst detectors suggests that our definition of bursts was largely similar to that of other authors.

There are several avenues through which this work could be extended. One area that we did not explore is the possibility of improving the results of burst detection by using a preprocessing step (Martens et al. 2014). Also, during our analysis, ideas arose about how the methods under review could be improved to enhance their performance. For example, for the CMA method, restricting the allowed values for maxISI to within a biologically realistic range may reduce the method's tendency to overestimate bursting in nonbursting spike trains and remove the need for post hoc screening. However, to ensure a fair and unbiased assessment of different burst detectors, we restricted our study to the original implementation of the authors' methods. Future studies in this area could look at how altering the existing methods could improve their performance.

Another area for consideration relates to which features of bursts are the most informative to extract. In past studies of rodent neuronal networks, we have shown that the temporal structure of bursting activity, measured by the CV of IBI, can be an important feature in distinguishing different types of network activity (Charlesworth et al. 2015). However, in the human network recordings examined here, we found no strong relationship between the CV of IBI and developmental age. A greater understanding of which are the most distinguishing features of bursts in human neuronal networks may inform future approaches to burst detection in these networks.

## GRANTS

Experimental data collection was supported by the Biotechnology and Biological Sciences Research Council (BBSRC) (P. Charlesworth, O. Paulsen; grant number BB/H008608/1). E. Cotterill was supported by a Wellcome Trust PhD Studentship and a National Institute for Health Research (NIHR) Cambridge Biomedical Research Centre Studentship. C. W. Thomas was supported by a bursary from the Bridgwater Summer Undergraduate Research programme.

## DISCLOSURES

No conflicts of interest, financial or otherwise, are declared by the author(s).

## AUTHOR CONTRIBUTIONS

E.C., P.C., O.P., and S.J.E. conception and design of research; E.C. and C.W.T. analyzed data; E.C. interpreted results of experiments; E.C. prepared figures; E.C., P.C., and S.J.E. drafted manuscript; E.C., P.C., O.P., and S.J.E. edited and revised manuscript; E.C., P.C., C.W.T., O.P., and S.J.E. approved final version of manuscript; P.C. performed experiments.
